# Effects of Myeloperoxidase-Induced Oxidation on Antiatherogenic Functions of High-Density Lipoprotein

**DOI:** 10.1155/2015/592594

**Published:** 2015-07-14

**Authors:** Takahiro Kameda, Ryunosuke Ohkawa, Kouji Yano, Yoko Usami, Akari Miyazaki, Kazuyuki Matsuda, Kenji Kawasaki, Mitsutoshi Sugano, Tetsuo Kubota, Minoru Tozuka

**Affiliations:** ^1^Analytical Laboratory Chemistry, Graduate School of Health Care Sciences, Tokyo Medical and Dental University, 1-5-45 Yushima, Bunkyo-ku, Tokyo 113-8519, Japan; ^2^Department of Laboratory Medicine, Shinshu University Hospital, 3-1-1 Asahi, Matsumoto 390-8621, Japan; ^3^Microbiology and Immunology, Graduate School of Health Care Sciences, Tokyo Medical and Dental University, 1-5-45 Yushima, Bunkyo-ku, Tokyo 113-8519, Japan

## Abstract

High-density lipoprotein (HDL) has protective effects against the development of atherosclerosis; these effects include reverse cholesterol transport, antioxidant ability, and anti-inflammation. Myeloperoxidase (MPO) secreted by macrophages in atherosclerotic lesions generates tyrosyl radicals in apolipoprotein A-I (apoA-I) molecules, inducing the formation of apoA-I/apoA-II heterodimers through the tyrosine-tyrosine bond in HDL. Functional characterization of HDL oxidized by MPO could provide useful information about the significance of apoA-I/apoA-II heterodimers measurement. We investigated the effects of MPO-induced oxidation on the antiatherogenic functions of HDL as described above. The antioxidant ability of HDL, estimated as the effect on LDL oxidation induced by copper sulfate, was not significantly affected after MPO oxidation. HDL reduced THP-1 monocyte migration by suppressing the stimulation of human umbilical vein endothelial cells induced by lipopolysaccharide (LPS). MPO-oxidized HDL also showed inhibition of THP-1 chemotaxis, but the extent of inhibition was significantly attenuated compared to intact HDL. MPO treatment did not affect the cholesterol efflux capacity of HDL from [^3^H]-cholesterol-laden macrophages derived from THP-1 cells. The principal effect of MPO oxidation on the antiatherogenic potential of HDL would be the reduction of anti-inflammatory ability, suggesting that measurement of apoA-I/apoA-II heterodimers might be useful to estimate anti-inflammatory ability of HDL.

## 1. Introduction

High-density lipoprotein (HDL) is known to have protective effects against the development of atherosclerosis by removing cholesterol from peripheral tissues following delivery to the liver and excretion in bile, a process called reverse cholesterol transport (RCT) [1, 2]. In addition, HDL has antioxidant ability—the ability to protect against the oxidation of low-density lipoprotein (LDL) [1, 3, 4]—and can inhibit monocyte migration to subendothelial space [1, 5, 6]. Therefore, structural changes in HDL would induce functional changes that could be followed by increased risk of cardiovascular disease.

One mechanism that could be connected with the formation of dysfunctional HDL is oxidation induced by myeloperoxidase (MPO) released from macrophages in atherosclerotic lesions. MPO catalyzes the chlorination or nitration of tyrosyl residues of apolipoprotein A-I (apoA-I) molecules in HDL [7–9]. When the tyrosyl residues of apoA-I and apoA-II are changed to radical form, the initial product of tyrosine oxidation by MPO [10], some apoA-I and apoA-II form heterodimers [11–14]. We previously observed that the levels of apoA-I/apoA-II heterodimers were significantly higher in plasma from patients with acute myocardial infarction compared to control patients [13]. Various reports have described the relationship between MPO-oxidized apoA-I and RCT. MPO was shown to reduce the RCT function of apoA-I [15–17], and apoA-I chlorination by MPO impaired ABCA1-dependent cholesterol transport [18]. However, the detailed mechanism by which MPO affects RCT by oxidation of apoA-I has not been fully elucidated [14, 19, 20]. In addition, the antioxidative potential of HDL containing apoA-I/apoA-II heterodimers is unknown. Therefore, we further investigated how oxidation of MPO affects the antiatherogenic properties of HDL, such as antioxidant ability, anti-inflammatory ability, and cholesterol efflux capacity. These results could improve our understanding of how quantitative estimates of MPO oxidation (e.g., by measurement of apoA-I/apoA-II heterodimers or by nitrated or chlorinated apoA-I) can reflect the pathogenesis of lesions.

## 2. Materials and Methods

### 2.1. Chemicals and Blood Samples

Unless otherwise stated, all reagents were purchased from Wako Pure Chemical (Tokyo, Japan). Blood samples were obtained from healthy volunteers who had provided informed consent. The study was approved by the ethics committee of Tokyo Medical and Dental University (number 1441).

### 2.2. Antibodies

Goat anti-human apoA-I and apoA-II polyclonal antibodies were purchased from Academy Biomedical (Houston, TX). Horseradish peroxidase- (HRP-) conjugated rabbit anti-goat IgG polyclonal antibody was purchased from Medical and Biological Laboratories (Nagoya, Japan). Biotinylated mouse anti-human apoA-I monoclonal antibody was purchased from Mabtech (Cincinnati, OH). Streptavidin-peroxidase was purchased from Sigma Aldrich Japan (Tokyo).

### 2.3. Isolation of Lipoproteins

LDL (1.006 < *d* < 1.063 g/mL) and HDL (1.063 <* d* < 1.21 g/mL) were isolated from pooled human serum by ultracentrifugation as described previously [21]. Isolated LDL and HDL were dialyzed against phosphate buffered saline (PBS) and stored at −80°C and 4°C, respectively, and HDL was used within 5 days.

### 2.4. Immunoblotting

Western blot analysis was performed as described previously [22]. Briefly, proteins separated by sodium dodecyl sulfate-polyacrylamide gel electrophoresis (SDS-PAGE), using 10–16% polyacrylamide gel under reducing conditions, were transferred to polyvinylidene fluoride (PVDF) membranes (Millipore, Bedford, MA). The membranes were incubated with anti-apoA-I and anti-apoA-II polyclonal antibodies, respectively, followed by incubation with HRP-conjugated rabbit anti-goat IgG. Finally, the bands containing apoA-I or apoA-II were visualized with 3,3′-diaminobenzidine tetrahydrochloride and hydrogen peroxide. The relative amounts of apoA-I/apoA-II heterodimer were analyzed with a CS Analyzer (ATTO, Japan).

### 2.5. Treatment of HDL with MPO

Five milliliters of HDL (10 mg protein/mL in PBS) was incubated with 5 mL of 50 mmol/L phosphate buffer (pH 7.4) containing 0.2 mmol/L of hydrogen peroxide and diethylenetriaminepentaacetic acid (DTPA) each, 0.4 mmol/L of l-tyrosine, and 20 nmol/L (unless otherwise stated) of MPO at 37°C for 1 hr and 24 hrs.

### 2.6. Antioxidant Ability

LDL oxidation induced by copper sulfate (CuSO_4_) was evaluated by the formation of conjugated dienes [23]. LDL (50 *μ*g protein/mL) was incubated with CuSO_4_ (1 *μ*mol/L) at room temperature in the absence or presence of HDL (50 *μ*g protein/mL), and the absorbance at 234 nm was monitored in 10 min intervals. The lag time was calculated from the kinetics as the intersection of the lag phase and the propagation phase lines [23]. Antioxidant ability of HDL was defined as relative prolongation of the lag time in the presence of HDL compared to LDL alone.

### 2.7. Cell Culture

THP-1, a human monocyte cell line, was obtained from ATCC (Manassas, VA) and propagated in RPMI 1640 at a density of 1 × 10^6^ cells/mL; the medium was supplemented with 10% fetal calf serum (FCS) containing penicillin (100 U/mL) and streptomycin (100 *μ*g/mL), and propagation took place at 37°C in 5% CO_2_. The cells were then washed extensively with serum-free RPMI 1640 medium, diluted to the appropriate density, and used in the experiments as indicated below. Cell viability exceeded 90%, as determined by trypan blue exclusion.

Human umbilical vein endothelial cells (HUVECs) were purchased from Lonza Japan (Tokyo) and cultured according to the manufacturer's instructions. Cells were cultured on gelatin-coated culture flasks in EGM-2 medium (Lonza) supplemented with 2% FCS. Confluent cultures at passage level 3 were washed with Hank's balanced solution, trypsinized, resuspended in complete media (as described above), and plated onto 24-well gelatin-coated plates at a density of 4 × 10^4^ cells/mL (500 *μ*L/well). After 48 hrs, cells were treated overnight with media containing 2% lipoprotein-deficient serum, which was separated from FCS as the bottom fraction (*d* > 1.210 g/mL) by ultracentrifugation. All incubations were performed in lipoprotein-deficient serum to minimize basal HUVEC activation.

### 2.8. THP-1 Cell Migration Assay

A THP-1 cell migration experiment was performed as described previously [24], using a Chemotaxicell containing polycarbonate filters (8 *μ*m pore size; Kurabo, Tokyo, Japan). The lower chambers were filled with 500 *μ*L of the conditioned media obtained from the postculture medium of HUVECs for 16 hrs at 37°C with or without lipopolysaccharide (LPS) (25 ng/mL) in the absence or presence of HDL (50 *μ*g/mL) and MPO-treated HDL (50 *μ*g/mL), of which monocyte chemotactic protein-1 (MCP-1) concentrations were measured using the ELISA kit (R&D Systems, Inc., USA). Then, the upper chambers were filled with 200 *μ*L of culture medium containing 2 × 10^5^ THP-1 cells. After 28 hrs of incubation at 37°C in 5% CO_2_, THP-1 cells that migrated to the lower chambers were counted on a Burker-Turk hemocytometer using an inverted microscope [25]. THP-1 cell migration was evaluated as the percentage of migrating THP-1 cells. The THP-1 cell migration rate in the absence of LPS and HDL was defined as 1.

### 2.9. HDL-Mediated Cholesterol Efflux from THP-1 Cells

THP-1 cells were grown in RPMI 1640, 10% FCS, 0.1% penicillin/streptomycin, and 0.1% nonessential amino acids at 37°C under 5% CO_2_. The cholesterol efflux studies were performed as previously described [26]. Three days prior to the experiment, cells were differentiated into macrophages by addition of phorbol 12-myristate 13-acetate (PMA: 100 ng/mL; Sigma). Then, macrophages were loaded with 50 *μ*g/mL acetylated LDL and 1 *μ*Ci/mL [^3^H]-cholesterol in RPMI 1640 containing 0.2% FCS. After 24 hrs, the labeling medium was removed and cells were washed three times with RPMI 1640 containing 0.2% FCS and then equilibrated in the same media for 18 hrs. Cholesterol efflux was assessed for 4 hrs using the media in the presence of 50 *μ*g/mL HDL, MPO-treated HDL, or no acceptor. Radioactivity in the media and total cell-associated radioactivity were determined by scintillation counting. The percentage of cholesterol efflux was calculated as [^3^H]-cholesterol in medium/([^3^H]-cholesterol in medium + [^3^H]-cholesterol in cells) × 100.

## 3. Results

### 3.1. Effects of MPO Treatment on HDL Structure

**Figure 1:**
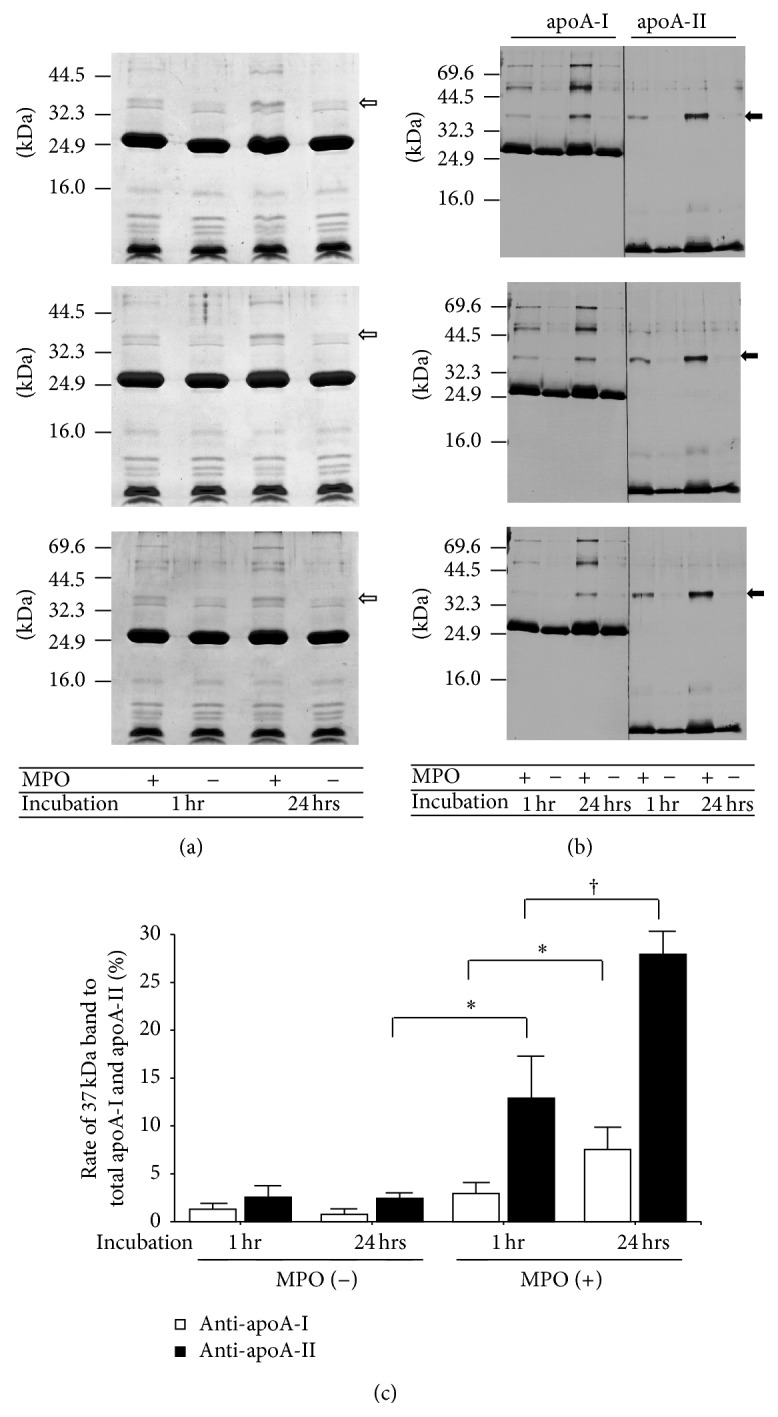
Apolipoprotein profiles of HDL treated with and without MPO. HDL treated with (+) or without (−) myeloperoxidase (MPO) for 1 and 24 hrs was subjected to SDS-PAGE followed by staining with Coomassie Brilliant Blue (a) and by immunoblotting for apoA-I and apoA-II (b). The 37 kDa bands, defined as apoA-I/apoA-II heterodimer, on CBB stained gel were indicated by white arrows. The bands that reacted with both anti-apoA-I and anti-apoA-II antibodies were identified as apoA-I/apoA-II heterodimers (black arrow). The molecular masses of standards are listed on the left. The ratios of apoA-I in apoA-I/apoA-II heterodimer to total apoA-I and apoA-II in apoA-I/apoA-II heterodimer to total apoA-II were estimated from immunoblotting patterns using a CS Analyzer (c). ^*∗*^ *P* < 0.05, ^†^ *P* < 0.01.

**Figure 2:**
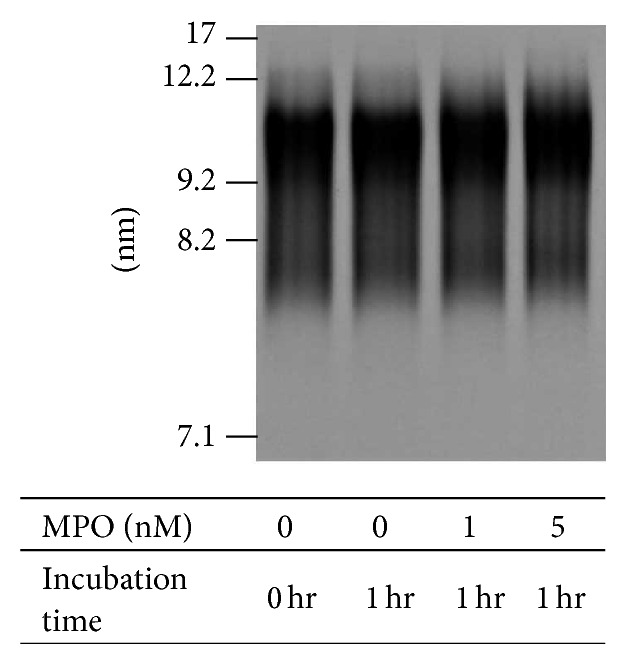
Effects of MPO treatment on HDL particle size. HDL treated with MPO (0, 1, or 5 nM) for 1 hr was subjected to nondenaturing PAGE followed by CBB R-250 staining. The particle sizes (nm) of the standards are listed on the left.

HDL treated with or without 20 nmol/L MPO for 1 and 24 hrs was analyzed by SDS-PAGE under reducing conditions using 10% gel, followed by staining with Coomassie Brilliant Blue R-250 (CBB) (Figure 1(a)) and by immunoblotting using anti-apoA-I and anti-apoA-II antibodies (Figure 1(b)). The CBB staining showed almost no differences in any HDL samples, except for 37 kDa bands, which tend to increase by MPO treatment. The 37 kDa bands were confirmed to react with both anti-apoA-I and anti-apoA-II antibodies and showed a tendency to be increased by MPO treatment in a time-dependent manner (Figure 1(c)). These bands appeared to be apoA-I/apoA-II heterodimers. apoA-I-containing complexes at approximately 50 and 80 kDa were also induced by MPO treatment. The bandwidth of apoA-I and apoA-II monomers (28 and 8.5 kDa, resp.) was slightly biased towards higher molecular mass after MPO treatment. MPO was also confirmed to have no apparent effects on HDL particle size on the native PAGE patterns (Figure 2).

### 3.2. Antioxidant Ability

**Figure 3:**
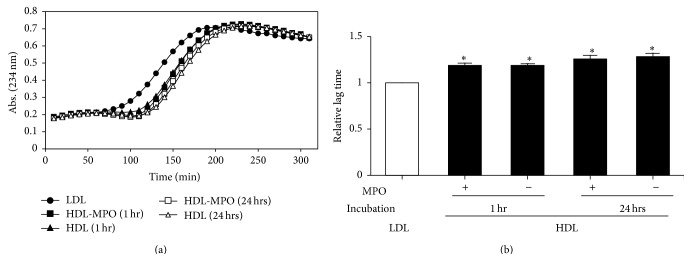
Antioxidant ability of HDL treated with and without MPO. LDL (50 *μ*g protein/mL) was oxidized by 1 *μ*M copper sulfate without (black circle) and with (other symbols) 50 *μ*g protein/mL HDL samples described in Figure 1. (a) Representative oxidation kinetics were obtained by monitoring diene formation at 234 nm at room temperature. (b) Relative lag times are shown (the lag time of LDL alone is defined as 1). Data represent the mean ± SD of three independent experiments. ^*∗*^ *P* < 0.01 versus LDL.

The kinetics of LDL oxidation by CuSO_4_ were analyzed in the presence of HDL treated with or without MPO for 1 and 24 hrs (Figure 3(a)). Although obvious differences between the presence and the absence of HDL were observed in the monitoring profiles, no significant differences were induced by MPO treatment of HDL. The lag times, which were used to quantitatively estimate antioxidant ability, were significantly prolonged by addition of HDL, at a similar rate compared to that of LDL alone (Figure 3(b)).

### 3.3. Effects of HDL on Monocyte Migration

**Figure 4:**
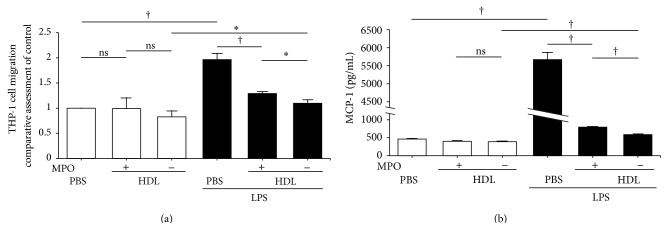
Effects of HDL treatment with and without MPO on THP-1 cell migration. The effects of HDL on THP-1 cell migration were determined using PET membranes (8 *μ*m pore size) (a). HUVECs were cultured in medium containing (black columns) or lacking (white columns) LPS with MPO-treated (+) and MPO-untreated (−) HDL or without HDL (PBS) at 37°C for 16 hrs. The media used for HUVEC culture were then supplied to a THP-1 cell migration assay. The THP-1 cell migration rate in the absence of LPS and HDL (PBS) was defined as 1. The results are means ± SD of three independent experiments. ^*∗*^ *P* < 0.05, ^†^ *P* < 0.01. ns: nonsignificant. MCP-1 concentrations in those media used for THP-1 cell migration assay were measured in triplicate by ELISA kit (b). The results are means ± SD of one experiment. ^†^ *P* < 0.01. ns: nonsignificant.

The culture medium of HUVECs stimulated by incubation with LPS at 37°C for 16 hrs in 5% CO_2_ accelerated the migration of THP-1 cells to a level twofold higher than that of the control (without LPS) (Figure 4(a)). However, the coexistence of MPO-untreated HDL with LPS suppressed the accelerated migration by 90%. In contrast, MPO-treated HDL exhibited a significant (70%) reduction in suppressive capacity compared to the untreated HDL. The culture medium of HUVECs containing only untreated HDL showed a tendency to suppress THP-1 cell migration compared to MPO-treated HDL without a statistical significance. It was also confirmed that LPS failed to have a direct influence on THP-1 cell migration (data not shown). MCP-1 concentrations in the culture media of HUVECs clearly reflected in the levels of migration of THP-1 cells (Figure 4(b)).

### 3.4. Cholesterol Efflux Capacity

**Figure 5:**
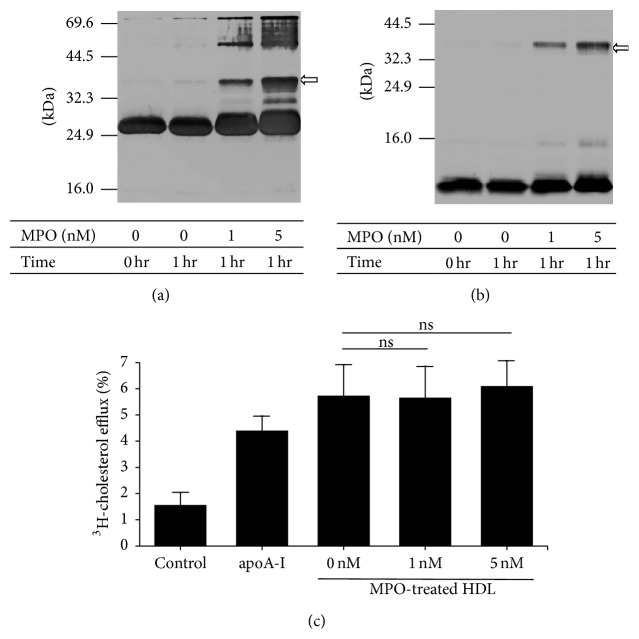
Effects of MPO treatment on HDL cholesterol efflux capacity. apoA-I/apoA-II heterodimer formation is induced by MPO oxidation. HDL treated with MPO (0, 1, or 5 nM) for 1 hr was subjected to SDS-PAGE followed by immunoblotting for apoA-I (a) and apoA-II (b). Arrows indicate the apoA-I/apoA-II heterodimer. Molecular masses of the standards are listed on the left. The amount of protein was 3 *μ*g/lane. [^3^H]-labeled cholesterol-loaded THP-1 macrophages were cultured in medium containing 50 *μ*g/mL HDL treated with 0, 1, or 5 nM MPO for 1 hr. Cholesterol efflux was determined after 4 hrs of incubation. Efflux media without HDL and with apoA-I (10 *μ*g/mL) were used as a negative control and for comparison, respectively. Results are means ± SD of three independent experiments. ns: nonsignificant.

HDL treated with MPO at 0, 1, or 5 nmol/L for 1 hr was analyzed by SDS-PAGE followed by immunoblotting using anti-apoA-I (Figure 5(a)) and anti-apoA-II (Figure 5(b)) antibodies. A dose-dependent increase in apoA-I/apoA-II heterodimer was confirmed. We incubated [^3^H]-labeled cholesterol-loaded THP-1 macrophages with this MPO-treated HDL to evaluate the capacity for cholesterol efflux (Figure 5(c)). Although SDS-PAGE profiles of apoA-I and apoA-II were apparently changed by MPO treatment, there was no effect of MPO treatment at any concentration on the cholesterol efflux capacity of HDL.

## 4. Discussion

SDS-PAGE followed by CBB staining and immunoblot analysis confirmed that treatment of HDL with MPO stimulated the generation of apoA-I/apoA-II heterodimers with an apparent molecular mass of 37 kDa. Bands of approximately 50 and 80 kDa, which showed higher intensities than the 37 kDa band by immunoblot using anti-apoA-I antibody, could be apoA-I dimers and trimers, respectively. Those dimers and trimers might have been formed by tyrosine-tyrosine bonds, since their molecular masses were not reduced by treatment with 2-mercaptoethanol. The intensity and width of apoA-I and apoA-II monomers in immunoblot patterns tended to increase and to spread, respectively, by MPO treatment. The increasedintensity probably indicates that the monomers were chlorinated by MPO treatment, followed by enhanced reactivity with their respective antibodies, as described previously [13, 27]. In addition, the slightly expanded width of the immunoblot bands induced by MPO treatment could indicate the increased molecular mass of the partial apoA-I and apoA-II by addition of tyrosine and/or chlorine molecules to tyrosine residues. It was also confirmed that MPO did not induce a reconstitution with obvious changes of HDL particle size.

No significant difference in antioxidant ability was observed between MPO-treated and MPO-untreated HDL. It means that apoA-I/apoA-II heterodimers in mature HDL particles did not have an impact on antioxidant ability, which probably indicated that a relatively small number of HDL particles contained apoA-I/apoA-II heterodimers under both physiological and forced oxidative conditions (Figure 1(b)) and that the lipids contained in HDL caused a structural reduction in the oxidative activity of the heterodimers. We previously reported that approximately 2% and 3% of total apoA-I and apoA-II, respectively, were present as the apoA-I/apoA-II heterodimer [13]. However, the possibility that nascent HDL, which contains extremely low amounts of lipids, might lose its antioxidant ability when oxidized by MPO is remained. In addition, enzymes attached to HDL, including LCAT and paraoxonase-1, were reported to prevent the oxidation of LDL [28, 29], suggesting that those enzymes—rather than apolipoproteins included in HDL—play a primary role in the antioxidant ability of HDL.

The present study demonstrated that HDL suppressed the stimulation of HUVECs induced by LPS, followed by the inhibition of monocyte chemotaxis. This activity could represent a part of anti-inflammatory property of HDL. Several studies have reported that HDL neutralizes the effects of LPS toxicity [30–32]. However, the suppressive ability of MPO-treated HDL was significantly reduced. These findings indicate that MPO released from macrophages partially impairs the anti-inflammatory ability, the neutralization ability in other words, of HDL. It might be connected with the differentiation to macrophages and release of MPO. These processes would take place in atherosclerotic plaques and precede atherosclerotic lesions, whereby macrophages are the first inflammatory cells to invade intima and are the main component of plaques [33].

It is known that stimulated endothelial cells release chemokines, such as MCP-1, fractalkine, and RANTES, and induce monocyte migration [34, 35]. In a basic study, recombinant MCP-1 directly induced THP-1 migration in a time-dependent manner, but the coexistence of HDL did not inhibit THP-1 cell chemotaxis (see Supplemental Figure 1 in Supplementary Material available online at http://dx.doi.org/10.1155/2015/592594). Our data suggested that HDL could act as a neutralizer against the stimulation of HUVECs by LPS followed by inhibition of MCP-1 secretion. In conclusion, untreated HDL showed a tendency to suppress THP-1 migration compared to MPO-treated HDL and PBS alone, indicating that MPO treatment reduces an anti-inflammatory potential of HDL through the neutralization of LPS.

In this cholesterol efflux study, 5 nM MPO-treated HDL showed a modest increase in cholesterol efflux capacity compared with untreated HDL; however, no significant differences were observed, despite the obvious change in apolipoprotein profiles on SDS-PAGE, which suggested that the formation of complexes including apoA-I and/or apoA-II in HDL did not affect cholesterol efflux capacity. Previous studies have suggested that the capacity of commercially available native human HDL to mediate cholesterol efflux was impaired by MPO modulation [36, 37]. On the other hand, cholesterol efflux capacity was enhanced by apoA-I/apoA-II heterodimers induced by oxidation of HDL by tyrosyl radicals [14]. It has also been reported that chlorination of apoA-I decreased cholesterol efflux capacity while nitration of apoA-I did not affect cholesterol efflux [18], suggesting that the molecular mechanisms responsible for cholesterol efflux capacity might be regulated by delicate structural changes in HDL apolipoproteins, especially apoA-I. It also cannot be denied that these differences were induced by the subculture conditions under which THP-1 cells were generated. A previous report indicated that the delicate culture conditions used for THP-1 cells can alter their response to stimulation, suggesting that discrepancies among results arise from differences among experiments [38].

Our results suggested that the oxidation of HDL by MPO caused significant reduction of anti-inflammatory potential. Therefore, quantitative assays of MPO-oxidized HDL might provide useful information about functional changes in HDL and indicate the levels of locally secreted MPO which reflect the state of atherosclerotic lesions. Although many researchers have attempted to use direct measurements of MPO in plasma as a biomarker of atherosclerosis [39, 40], we suggest that a marker such as the apoA-I/apoA-II heterodimer, which would reflect MPO activity limited to atherosclerotic lesions, is a better indicator of the atherosclerotic state than plasma MPO activity. The plasma MPO activity might not be necessarily proportional to the functional reduction of HDL. This indicates the importance of measuring the materials generated by MPO (e.g., apoA-I/apoA-II heterodimers and nitrated and chlorinated apoA-I) as markers that reflect the atherosclerotic state, in connection with macrophages, LDL, and HDL in the lesion.

Further studies are needed to elucidate the actual mechanism behind the contribution of MPO to the development of atherosclerosis and to assess the potential of apoA-I/apoA-II heterodimers as a biomarker for estimating the anti-inflammatory ability of HDL.

## 5. Conclusion

The principal effect of MPO oxidation on the antiatherogenic potential of HDL would be the reduction of anti-inflammatory ability. It suggests that measurement of apoA-I/apoA-II heterodimers, which would reflect MPO activity limited to atherosclerotic lesions, might be useful to estimate anti-inflammatory ability of HDL.

## Supplementary Material

The recombinant MCP-1 directly induced THP-1 cell migration in a time-dependent manner. However, the coexistence of HDL did not significantly inhibit THP-1 cell chemotaxis, indicating that HDL neutralized LPS and partially inhibited the secretion of MCP-1 from HUVECs, but not directly affected MCP-1 function in THP-1 cell migration. It was also confirmed that HDL itself did not induce THP-1 cell migration.
