# Supplementary figures and images for: Effects of Myeloperoxidase-Induced Oxidation on Antiatherogenic Functions of High-Density Lipoprotein
Source: J Lipids. 2015 Jul 14;2015:592594. doi: 10.1155/2015/592594 (PMC4516847; doi:10.1155/2015/592594)

Supplement Figure 1

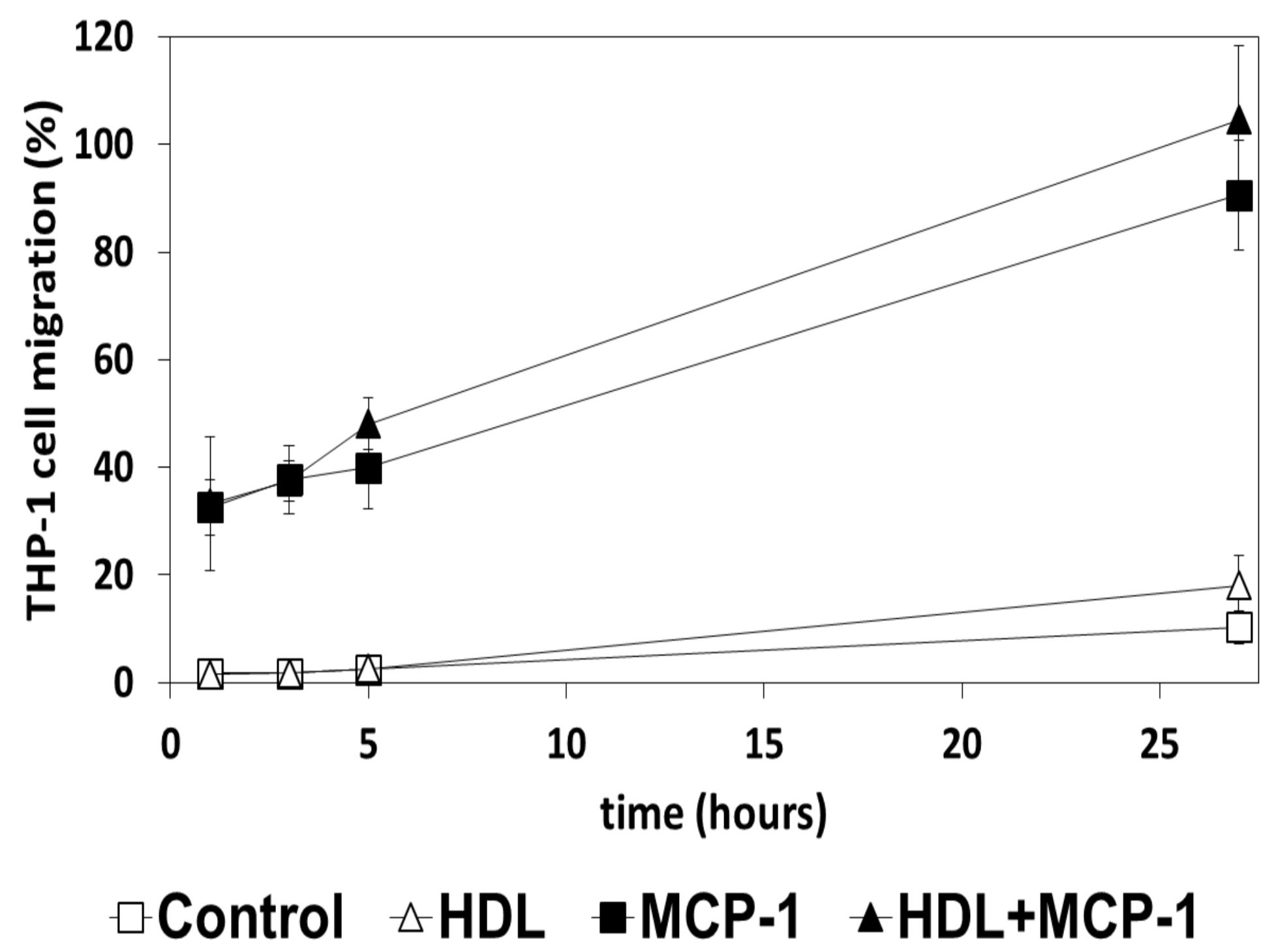

Supplement: Supplementary file 1 — The recombinant MCP-1 directly induced THP-1 cell migration in a time-dependent manner. However, the coexistence of HDL did not significantly inhibit THP-1 cell chemotaxis, indicating that HDL neutralized LPS and partially inhibited the secretion of MCP-1 from HUVECs, but not directly affected MCP-1 function in THP-1 cell migration. It was also confirmed that HDL itself did not induce THP-1 cell migration. [file 592594.f1.pdf]
